# Explanatory definition of the concept of spiritual health: a qualitative study in Iran

**Published:** 2018-04-09

**Authors:** Ahmad Ghaderi, Seyed Mahmoud Tabatabaei, Saharnaz Nedjat, Mohsen Javadi, Bagher Larijani

**Affiliations:** 1 *PhD Candidate in Medical Ethics, Medical Ethics and History of Medicine Research Center, Tehran University of Medical Sciences, Tehran, Iran; Department of Medical Ethics, School of Medicine, Tehran University of Medical Sciences, Tehran, Iran.*; 2 *Professor, Medical Ethics and History of Medicine Research Center, Tehran University of Medical Sciences, Tehran, Iran.*; 3 *Professor, Epidemiology & Biostatistics Department, School of Public Health, Tehran University of Medical Sciences, Tehran, Iran.*; 4 *Professor, Department of Ethics, Qom University, Qom, Iran.*; 5 *Professor, Endocrinology and Metabolism Research Center, Endocrinology and Metabolism Research Institute, Tehran University of Medical Sciences, Tehran, Iran. *

**Keywords:** *Spiritual health*, *Spirituality*, *Health-care ethics*

## Abstract

Scientists and researchers have examined spiritual health from different angles and proposed various definitions, but a comprehensive definition does not exist for the term as of now. The present study aimed to offer the definition, components and indicators of spiritual health from experts’ perspective.

This qualitative study utilized conventional content analysis and individual in-depth interviews with 22 experts in the area of spiritual health in various fields selected through purposeful sampling. Member check, credibility, reliability, transferability and allocation of adequate time for data collection were measured to increase the validity and reliability of the results. Conventional content analysis was performed in three main phases: preparation, organization and reporting, and the categories, subcategories and codes emerged accordingly.

Participants defined spiritual health in three dimensions: religious, individualistic, and material world-oriented. The study revealed four types of connection in spiritual health: human connection with God, himself, others and the nature. The majority of participants stated that spiritual health and spirituality were different, and pointed out the following characteristics for spiritual health: it affects physical, mental, and social health; it dominates other aspects of health; there are religious and existential approaches to spiritual health; it is perceptible in people’s behavior; and it can be enhanced and improved. Most experts recognized human connection with God as the most important part of the definition of spiritual health. In conclusion, the connection between humans and themselves, others and the nature was not seen as a component specific to spiritual health.

## Introduction

The word “spirit” is derived from the Latin words “spiritus” (meaning breath, courage, vigor, or soul) and the word “spirare” (meaning to breathe) (1). Five characteristics of spirituality include: meaning, value, transcendence, connecting (with oneself, others, God/supreme power and the environment), and becoming (the growth and progress in life) (2).

Five decades have passed since the introduction of spiritual health and its various definitions. Spiritual health is about the connection with self (personal dimension), others (social dimension), the nature (the environment) and God (transcendental dimension) (3). The basic characteristics of spiritual health are as follows: proper lifestyle, connection with others, asking about the meaning and purpose of life, and transcendence (4). Spiritual health is extremely important for many researchers, to the extent that it is seen as one of the key aspects of health (5). According to numerous studies conducted on various patients, spiritual health leads to improved mental health (6), and is positively related to physical health, for instance, it may help patients experience lesser pain (7). 

Scientists and researchers have studied spiritual health from different angles and offered various definitions for it, but have failed to propose a comprehensive definition for the term. Major challenges for addressing issues related to spiritual health include: providing a comprehensive definition for it; identifying the components and indicators of spiritual health; and its effect on other aspects of health. Even though a number of studies have been conducted on the subject of spiritual health in Iran (5, 7), there are not enough studies on the definition of the term according to experts. Given the importance of the issue, the shortage of literature, and the need to consider different views about spiritual health, this study was conducted in Iran to explore the definition, components and indicators of spiritual health from experts’ perspective.

## Method

This was a qualitative study in which we arranged individual in-depth interviews with experts and analyzed the contents. Experts in the area of spiritual health in various fields such as ethics, philosophy, theology, medicine, psychiatry, psychology, health and medical ethics were selected by purposeful sampling. The experts were academicians, authorities on health-care system in the Ministry of Health, researchers and authors in the field of spiritual health and spirituality, and members of the Spiritual Health Department in the Academy of Medical Sciences of Iran. The selected experts had articles, books or lectures in the field of spiritual health. It should be noted that other qualified people in the field of spiritual health were also consulted who were contributed by selected experts. After explaining the objectives of the study, observing confidentiality and obtaining informed consent from participants, individual semi-structured in-depth interviews were conducted until data saturation. A total of twenty-two interviews were conducted, each lasting between 40 to 50 minutes. The time and location of interviews were determined by the experts. Interview questions included: “What is your definition of spiritual health?” “What are the components of spiritual health?” “What are the indicators of spiritual health?” “Do you think spiritual health and spirituality are different or not?” and “Please mention other points, if any”. A voice recorder was used to record the interviews, and notes were taken of the experts’ talks. The experts were informed that they could ask the researcher to pause the recording anytime during the interview, or withdraw from the study at any stage. The experts’ responses were returned to them to check in order to increase the validity and reliability of the study (member check). Other measures that were used to increase the validity of the results included credibility, reliability, transferability and allocation of adequate time for data collection. Data analysis was performed through conventional content analysis in three main phases: preparation (selecting the unit of analysis, immersion in the data); organizing (coding, creating categories and abstraction); and reporting. The contents of the interview audio files were transcribed and the transcripts were carefully and repeatedly read to extract meaning units, which could be either words or sentences. Thus open codes were extracted, redundant codes were eliminated, and the codes that propounded a single subject were placed in one category. Next, key and abstract concepts were extracted, and categories, subcategories and codes emerged. 

## Results

The results of the study showed five clusters of information about spiritual health including its definition, components, indicators, characteristics and the differences between spiritual health and spirituality. Each cluster was characterized by specifications as presented in Table 1.

**Table 1:** The results in the form of categories and subcategories

**Category**	**Subcategory**
Definition of spiritual health	Religious dimension Individualistic dimension Material world-oriented dimension
Components of spiritual health	Religious (Human connection with God) Individualistic (Human connection with himself) Material world-oriented (Human connection with others and the nature)
Indicators of spiritual health	Human connection with God Human connection with himself Human connection with others Human connection with the nature
Differences between spiritual health and spirituality	Relative coincidence between spiritual health and spirituality
Characteristics of spiritual health	Different definitions for spiritual health The effect of spiritual health on the other three dimensions of heath The dominance of spiritual health over other aspects of health The religious and existential approaches to spiritual health The manifestation of spiritual health in behavior Spiritual health as a dynamic process

**Definition of Spiritual Health**

Participants believed that spiritual health has three dimensions: religious, individualistic, and material world-oriented. 

*Religious Dimension:*

According to the participants, this dimension of spiritual health entails knowledge, attitude and practice based on divine unity so that one has a dynamic and active relationship with oneself, others and the nature because one considers God in all connections. In this regard, one of the participants said, “*Spiritual health means to move toward God*”. 

*Individualistic Dimension:*

The study participants indicated that this dimension may be defined as:

1) Believing in the meaning and spirituality, and absence of spiritual ailments such as hopelessness and lack of love, happiness, forgiveness and common sense experiences in one’s interactions with others. One participant said, “*The minimum level of spiritual health means that an individual does not suffer from spiritual ailments and has embraced spirituality*”.

2) Having good moral character and decent beliefs. This specification can be noted in one participant’s words: “*A person who enjoys spiritual health does not have negative moral characteristics and wrong beliefs*”.

3) Parts of health or human existence that cannot be explained from physical, mental or social perspectives. Spiritual health includes a purposeful life, transcendence and actualization of different dimensions and capacities of human beings. Spiritual health creates a balance between physical, psychological and social aspects of human life. Some participants presented their perception of spiritual health as follows:

*“* *Those components of health that are not physical, psychological or social can be placed in the context of spiritual health* *”.*

*“* *Spiritual health promotes human capacity* *”.*

*“* *Spiritual health means a purposeful life* *”.*

*“* *Spiritual health causes a balance between internal human possibilities* *”.*

4) Individuals’ feelings about a supreme power, themselves and others; having positive feelings; balance; peace; feeling vitality and empowerment, hope and satisfaction; and reduced unpleasant feelings such as sadness, anxiety and anger. One of the interviewees stated, “*I think an important parameter of spiritual health is to have positive feelings about spiritual matters*”.

*Material World-Oriented Dimension:*

This dimension is defined as well-wishing, helping others without expecting them to return favors, feeling of closeness and harmony with the universe, and human connection with himself, others and the nature based on moral virtues. With respect to the individualistic dimension, one interviewee said, “*Spiritual health is helping others without expecting something in return*”.

Additionally, participants stated that spiritual health has different definitions based on individuals’ beliefs and opinions. According to one participant: “*Spiritual health is as varied as people’s perspectives, and therefore it is impossible to offer a single definition for spiritual health*”.

**Components of Spiritual Health**

According to the results of our study, the three components of spiritual health are: religious, individualistic, and material world-oriented.

*Religious Component*

According to our findings, the religious components may be the result of human connection with God, including a sense of connection with God, love of God, prayer, the feeling that God is effective, and God-oriented knowledge, attitude, and behavior. As one of the participants mentioned, “*The most important and essential component of spiritual health is communication with God*”.

*Individualistic Component*

The individualistic component comes from human connection with himself. Instances include: self-scrutiny, examining the meaning of life, hope, self-actualization, moral virtues, peace, responsibility for oneself, balance, transcendence, values, mysticism, culture, and knowledge, attitude, and behavior in relation with oneself.

In this regard a participant stated, “*The second component of spiritual health is the humans’ connection with themselves*”.

*Material World-Oriented Component:*

This refers to human connection with others and the nature*. *Connection with others creates a sense of responsibility toward them, unconditional love, forgiveness, pacifism, harmony with others, and knowledge, attitude, and behavior in relation with others.

Human connection with the nature develops responsibility, love, and knowledge, attitude and behavior in relation with the nature.

To quote a participant: “*One component of spiritual health is that man should form his relationship with others and with the nature*”.

**Indicators of Spiritual Health**

According to our participants, the indicators of spiritual health entail four domains of the connection between humans and God, themselves, others, and the nature.

*Human connection with God:*

The connection with God is characterized by knowing God, feeling affection and love toward God, laying one’s hopes on God, being thankful for divine blessings, and prayer. These specifications were reflected in one of the participants’ perception of God as follows: “*The first and most important indicator of spiritual health is obeying God’s commands”*.

*Human connection with himself:*

This type of connection is a source of self-esteem, reasoning and thinking, calmness, responsibility for oneself, satisfaction, vitality, empowerment, hope, a sense of purpose, self-worth, and self-awareness.

One of the interviewees stated, “*The second indicator of spiritual health is rationality in personal and social behaviors*”.

*Human connection with others:*

The participants believed that connection with others could positively affect an individual’s behavior and result in acceptance of social responsibility, respect for the rights of others, honesty, compassion, altruism, generosity, optimism, empathy, benevolence, helping others unconditionally, humility, and lack of jealousy and grudge.

One participant stated, “*The indicators of spiritual health are human behaviors, for instance the sense of responsibility toward others*”.

*Human connection with the nature:*

The study participants presumed certain behaviors to be the result of human connection with the nature. Instances included recognizing one’s duties in dealing with the nature, acknowledging the importance of interest in the nature, and showing respect for it.

**Differences between Spiritual Health and Spirituality**

The participants’ views on the differences between spiritual health and spirituality can be divided into two categories: 

1) The majority of our participants believed that spiritual health and spirituality are different because: a) spirituality is a state of being, but spiritual health is a state of having; b) spirituality is a general concept, while spiritual health is a particular concept; c) spirituality is a subjective issue, but spiritual health is objective; d) spirituality is a comprehensive issue, and spiritual health is the subset of spirituality; e) spirituality is potential, but spiritual health is actual.

Accordingly, one participant said, “*If you want to define spiritual health in medical literature, it is quite different from spirituality in the sense of value-judgment*”.

2) Some were of the opinion that there is a relative coincidence between spiritual health and spirituality, and that the former is the manifestation (product) of the latter.

One of the participants remarked, “*If we define spirituality as whatever leads humans to perfection and happiness, then people who are more spiritual will be healthier*”.

**Characteristics of Spiritual Health**

According to our participants, spiritual health has 6 different characteristics: 

1**) **It has different definitions.

2) It affects physical, mental, and social health. 

3) It is preferred over the other aspects of health. 

4) There are religious and existential approaches to it. 

5) It is presented in individuals’ behavior. 

6) It is a dynamic state and can be promoted.

The following statements also reflect the characteristics of spiritual health according to some participants:

“*The definition of spiritual health varies based on the views of individuals*”. 

“*Spiritual health can affect other aspects of health, meaning physical, mental, and social health*”.

“*Spiritual health encompasses physical and mental health*”.

“*There are different approaches to spiritual health: one is the religious approach and another is the existential approach*”.

“*Spiritual health is manifested in the behavior of people*”.

“*Spiritual health can be improved*”.

## Discussion

Our study revealed that in order to approach spiritual health, we need to consider five clusters of information: its definition, components, indicators, characteristics and the differences between spiritual health and spirituality. Each cluster is discussed separately as follows: 

*Definition of Spiritual Health:*

According to the existing literature, spiritual health is the connection with God (a superior existence), oneself, others and the nature (3, 8). Likewise, our participants’ responses indicated four types of connection in spiritual health, that is, human connection with God, himself, others and the nature. The participants believed that spiritual health encompasses those features of health or human existence that cannot be explained from physical, mental and social aspects. Absence of spiritual ailments is embedded in the definition of spiritual health.

Based on our participants’ opinions, a suitable human connection with oneself, others and the environment reflects mental and social health, and is therefore not specific to spiritual health. In response to the claim that spiritual health means absence of spiritual ailments, it should be mentioned that these ailments need to be precisely defined. Moreover, we need to develop tools to determine spiritual ailments, and then ascertain the absence of which ailments and to what degree would indicate spiritual health, which is a rather difficult thing to do.

*Components of Spiritual Health:*

In agreement with the literature, our results show that spiritual health is determined by the components of connection with oneself, God (transcendent reality), society (others), and the nature (9, 10). 

*Indicators of Spiritual Health:*

Studies show that spirituality is a way through which one communicates with oneself, others, the nature and everything that is sacred (holy entities) (11, 12).

It seems that the indicators of spiritual health can be investigated in four domains, that is, human connection with God, himself, others, and the nature.

As previously explained, human connection with himself others and the environment are not specific to spiritual health; therefore, it can be stated that the above-mentioned components and indicators also exist in the domain of mental and social health. 

*Differences between Spiritual Health and Spirituality:*

There is a consensus among researchers that spiritual health be treated as a sub-concept of spirituality (13). After reviewing the literature, Selman et al. concluded in 2007 that spirituality has six aspects and indicators of spiritual health, for instance peace and a sense of control are among the aspects of spirituality (14).

It seems that spiritual health and spirituality are different, although there is a relative coincidence between the two and they overlap.

*Characteristics of Spiritual Health:*

Several definitions and characteristics were identified for spiritual health (15). In any given culture, spirituality has different meanings (16), which is in agreement with our findings that produced various definitions and characteristics for spiritual health based on individuals’ beliefs and worldviews.

A large number of systematic reviews showed that spiritual health can contribute to positive health outcomes (17). Heidari et al. revealed that spiritual health improves physical well-being and quality of life (18). Research conducted on patients with asymptomatic heart failure in 2009 indicated that spiritual health is positively related to better mental health (19). Rahnama et al. also showed that spiritual health prevents the emergence of depression and anxiety in patients with spinal cord injury (20). This is in agreement with the results of the present study that illustrated the positive effect of spiritual health on physical, mental and social health.

Literature review showed that spiritual health unites the physical, mental and social dimensions of people (21). Moreover, some health models consider spiritual health the focus or the most significant of all other aspects of health, which emphasizes the importance of spiritual health (1).

Based on our participants’ responses, it can be stated that spiritual health encompasses other dimensions of health and is more important than its other aspects.

However, in interpreting the results we should consider the type of the studies, because cross-sectional studies cannot determine the casual relationship, but measure the correlation between two variables.

Spiritual health has two dimensions: religious and existential. Religious health is related to individuals’ perception of their health in relation to a supreme power, while existential health is related to their socio-mental preoccupations and their way of interacting with themselves, the society and the nature (5). Spiritual well-being has two components: religious well-being, which refers to the connection with God or a higher being, and existential well-being, which pertains to the meaning and purpose in life (22).

Accordingly, it can be assumed that spiritual health has two dimensions: religious health, which is shaped in connection with God or a supreme power (a transcendent and holy existence), and existential health in human connection with himself, others and the nature, the meaning and purpose in life.

People with better spiritual health are more likely to select a lifestyle that enhances their health (17). An appropriate lifestyle is a fundamental characteristic of spiritual health (4). Likewise, it can be concluded that spiritual health presents itself in the individual’s behavior.

Spirituality is a dynamic state; it can be promoted, its identities and patterns may change, and it can be hidden or invisible (23). Our study participants believed that spiritual health has a dynamic nature, i.e., it can be increased or decreased.

In conclusion, the present study confirmed four types of connection in the definition of spiritual health: human connection with God, himself, others and the nature. Most of the experts who participated in the current study recognized human connection with God as the most important part of the definition of spiritual health and also stated that human connection with God is the most important component and indicator of spiritual health. Other studies, on the other hand, could not show the superiority of one component over the others. Human connection with himself, others and the nature is not specific to spiritual health because these components of spiritual health are present in mental and social health as well. Although we found different definitions for spiritual health, we believe that these may differ based on individuals’ beliefs and opinions. It is suggested that further longitudinal studies be conducted to provide more accurate explanations for spiritual health by interdisciplinary collaboration between mental health specialists, social health specialists and religious scholars. Such studies would result in more accurate research on spiritual health and its causal relationship with physical health, mental health and social health.
